# Infrequent, but Not Intricate Radiological and Pathological Diagnosis of Chronic Intestinal Pseudo-Obstruction—Presented in a Two Pediatrics Cases of the Visceral Myopathy

**DOI:** 10.3390/diagnostics15192503

**Published:** 2025-10-01

**Authors:** Monika Kujdowicz, Grażyna Drabik, Damian Młynarski, Katarzyna Jędrzejowska, Wojciech Górecki, Anna Wierdak, Kamila Płachno, Józef Kobos

**Affiliations:** 1 Department of Pathomorphology, Jagiellonian University Medical College, Grzegórzecka 16, 31-531 Krakow, Poland; 2 Department of Pathomorphology, University Children’s Hospital of Krakow, Wielicka 265, 30-663 Krakow, Poland; 3 Department of Pediatric Surgery, Institute of Pediatrics, Jagiellonian University Medical College, Wielicka 265, 30-663 Krakow, Polandwojciech.gorecki@uj.edu.pl (W.G.); 4 Nutritional Treatment Department, University Children’s Hospital of Krakow, Wielicka 265, 30-663 Krakow, Poland; 5 Department of Pathomorphology Central Clinical Hospital, Medical University of Lodz, Pomorska 251, 92-213 Lodz, Poland; jozef.kobos@umed.lodz.pl

**Keywords:** visceral myopathy, smooth muscle actin, fibrosis, constipation, chronic intestinal pseudo-obstruction, Hirschsprung disease

## Abstract

Obstruction differential diagnosis involves tumors, “acute abdomen”, and chronic pseudo-obstruction (CIPO). Pediatric CIPO cases have different backgrounds than adults’ and impairs development. The cases are rare; diagnosis and treatment are still not well established. Diagnosis is complex; clinical, radiological, molecular, and manometric pathologic data are essential. The performance of broad radiological investigations and manometry is cumbersome in a small intestine. Herein, we present cases of a 14-year-old girl and 11-year-old boy with visceral myopathies (VMs). Presented cases show unique hardship in the analysis of standing and contrast bedside X-ray images—the colon distension alone speaks to Hirschsprung, and the clinicians could not confirm suspected short-segment disease for a long time. VMs are usually diagnosed up to 12 months of life and accompanied by other organ dysfunctions, which are herein absent. The key features here were also the involvement of the small intestine, lack of distant colon contraction, and for the long-lasting case in the boy, loss of haustration. The initial diagnosis relied on clinical data (vomiting, malabsorption, >6-month obstruction, and uncharacteristic biochemical tests), radiology (lack of tumor, enlargement of diameter, and fluid in small and large intestines), and manometry (presence of propagation wave and of anal inhibitory reflex in recto–anal manometry). Examination of intestinal muscle biopsies involved hematoxylin-eosin, trichrome-Masson staining, and immunohistochemistry. The characteristics were fibrosis, small vacuoles, muscle layer thinning, and decreased expression of smooth muscle actin and desmin. The localization of biopsies was chosen after X-ray examination, due to interruption and with various degree changes. The final diagnosis was put forward after the analysis of all accessible data. The diagnosis of VM underlines the importance of interdisciplinary co-work. An earlier intestine muscle biopsy and well-designed molecular panel might fasten the process of diagnosis. Deeper exploration of phenotype–genotype correlation of various VM presentations in the future is crucial for personalized treatment.

**Figure 1:**
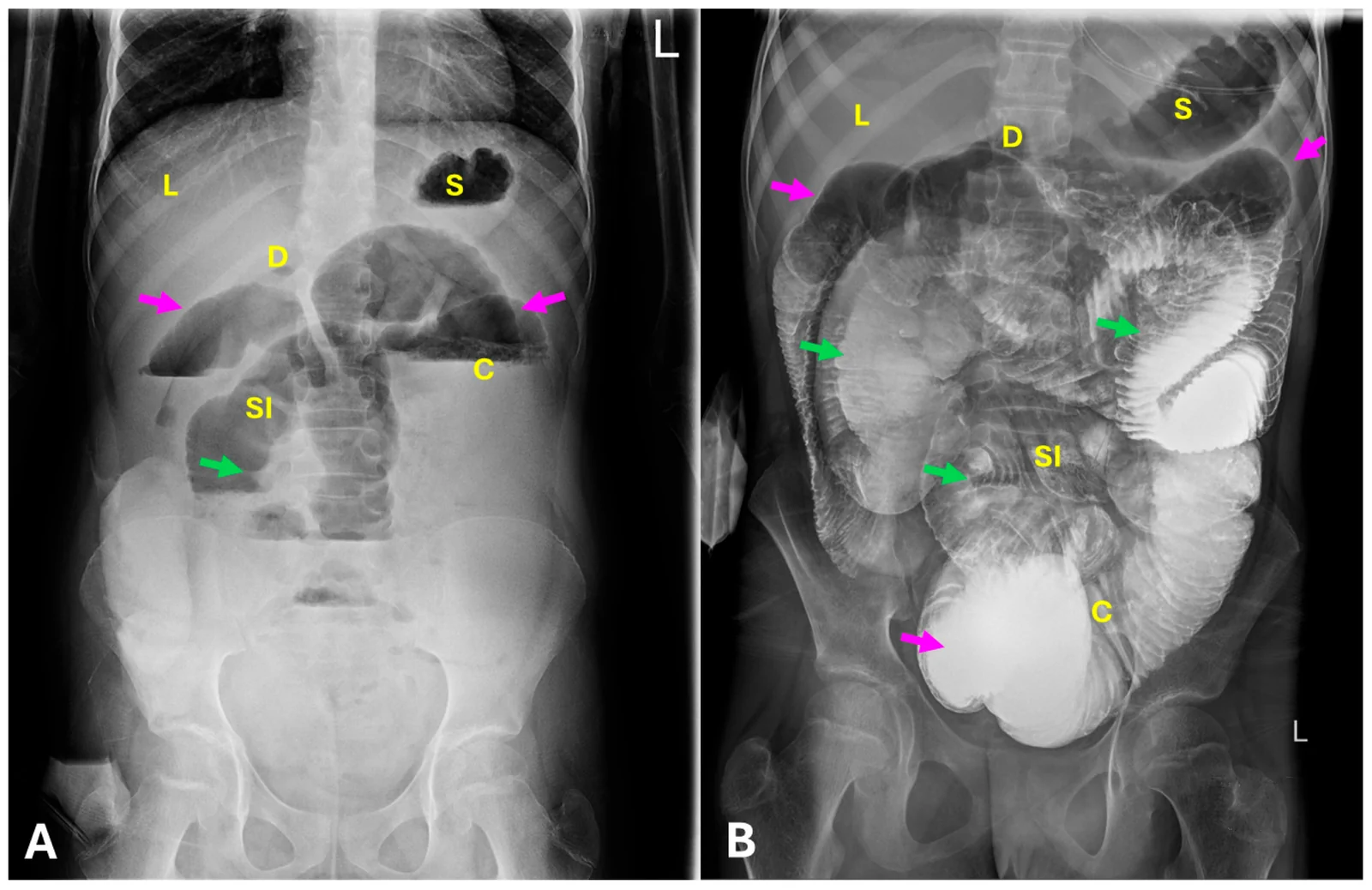
Radiological findings in the 14-year-old girl. (**A**)—standing X-ray; (**B**)—contrast bedside X-ray. S—stomach; D—duodenum; SI—small intestine; C—colon; L—liver. Arrows: magenta—smooth intestine borders (loss of haustration and folds); green—thickened folds. The girl was diagnosed with intestinal motility disorders, constipation, and malabsorption from birth, also depressive episodes. She had a three-year history of progressive abdominal distension, feeding intolerance, bloating, and abdominal pain. CT and MRI imaging of the intestines performed at age 12 showed no abnormalities. Upon admission, she was in moderate general condition with severe cachexia (BMI 12.3), a midline laparotomy scar (~15 cm), and two stomias. Parenteral nutrition had been administered (6.6 Fr central catheter). She was consulted by a surgeon, geneticist, psychiatrist, dietitian, ophthalmologist, neurologist, and immunologist. A broad spectrum of serum and stool tests had been performed. Since February 2024, she underwent five abdominal surgeries for paralytic and adhesive obstructions, including resection of approximately 10 cm of the small intestine and the formation of an ileostomy (10–15 cm proximal to the ileocecal valve) and colostomy. Despite surgical interventions, symptoms persisted. The patient remained dependent on parenteral nutrition and tolerated only minimal oral feeding. Abdominal radiography showed massively dilated bowel loops with multiple air–fluid levels (**A**). Barium contrast X-ray (fluoroscopy) analysis excluded other causes of obstruction, such as tumor, lymphocytic hyperplasia, hamartoma, duplication, ulcer, and the like [1]. A contrast study revealed small intestine loops dilated over a long segment/along their entire length, with contraction occurring only in a short proximal segment (duodenum) (**B**). Herein, dilatation was present in the small and large intestine but absent in the other parts of the digestive tract (e.g., esophagus, stomach), as well as in other organs such as the bladder. The symptoms and imaging were enigmatic, as many diseases can present similarly. The images evoked suspicion of Hirschsprung disease, and in re-analysis after muscle biopsy, also myopathy [2].

**Figure 2:**
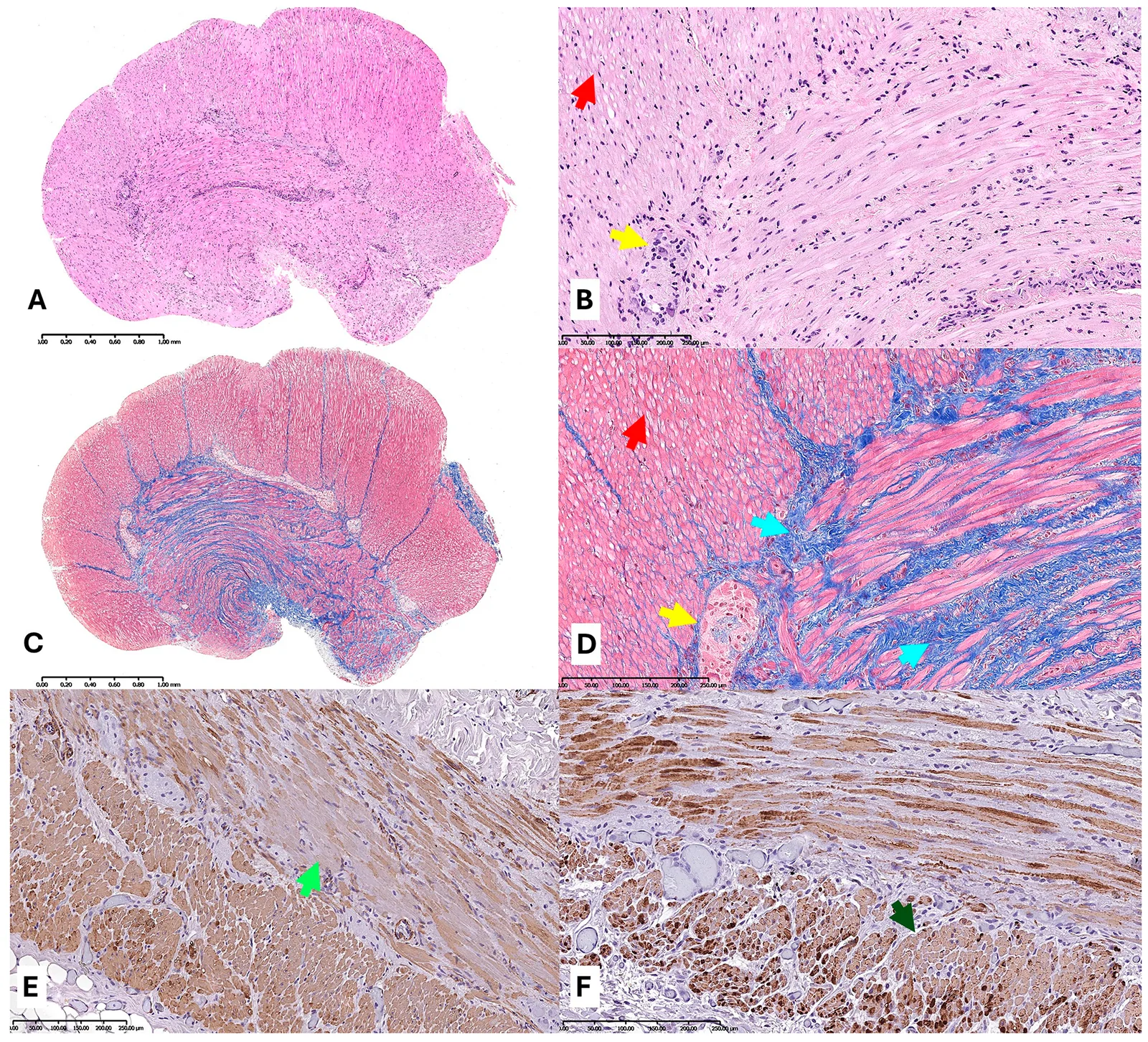
Microphotographs of the rectum biopsy (**A**–**D**) and ileal fragment (11.5 cm length—resected due to dilatation and purulent exudate, which increases the risk of perforation; (**E**,**F**)). (**A**,**B**). Hematoxylin-eosin (HE; ×10, ×100). (**B**,**C**). Trichrome-Masson (TM; ×10, ×100). (**C**). Smooth muscle actin (SMA; ×200). (**D**). Desmin (×200). Arrows: yellow—ganglion cells of the Myenteric plexus; red—vacuoles; cyan—fibrosis, green—decreased SMA and desmin expression, bright and dark shades, respectively. The 14-year-old girl was consulted in our hospital in June 2024. Diagnostic ileal mapping biopsies of muscularis propria were taken from the peritoneal recess and 10 cm above, the descending colon, the splenic flexure, the transverse colon, the rectum, and the ileum. Full-thickness biopsies are recommended only for patients with laparoscopy and surgery. HE staining revealed the presence of ganglion cells in the intramuscular nerve plexuses, partial thinning of both muscle coats, and the brightening of some myocytes (vacuoles) (**A**,**B**). On that step, differential diagnosis involved inflammatory diseases (mainly systemic sclerosis, but Scl-70 was negative), and neuromuscular disorders. Laboratory tests did not indicate any immunological or metabolic disorders [3,4]. The manometry outcomes excluded neurological background. HE staining did not reveal mitochondrial clumps (could be seen as red dots near nucleus, usually in ganglion cells). In and around the nerve plexuses there are, at most, very few single leukocytes (CD45 positive). Lack of inflammation excluded infections. Lack of hyalinization excluded amyloidosis. The relative thickness of muscle coats varies with individual patients, age, and anatomic location. The most probable cause was myopathy, according the data above. HE staining revealed variation in nuclear and fiber size, focal changes, various intensity of staining, and cytoplasmic PAS-negative vacuoles (called M-bodies), these are helpful for the differential diagnosis of storage diseases. Vacuoles are present in various cases of slow transit and diabetic gastropathy, they also could be artifacts [5,6,7]. TM staining showed fibrosis of the muscle membrane (seen as blue connective tissue areas between stained red hypotrophic muscle fibers) [8,9]. Here, in some parts, fibrosis was more severe in the inner muscle coat, in others, it was similar in both coats (**C**,**D**). SMA and desmin were almost normal, but in some cells the expression was greater, and in some it was very little. VM involves muscularis propria malformations of the enteric coat, muscle cell degeneration, and filament protein abnormalities. In these, the intestinal muscle is overstretched, and between the cells there is the production of connective tissue, which in turn produces fibrosis. The overstretch is caused by a lack of tethering proteins and large, easy-breaking gaps between muscle cells. Finally, the examination of DOG1 excluded Cajal cell dysfunction [10]. Whole exome sequencing did not reveal any mutations corresponding to the reported clinical symptoms [11]. Before diagnosis, the patient exhibited behavioral disturbances, such as attempts to detach the central venous catheter, and food-seeking behaviors. Following the confirmation of her diagnosis and psychiatric intervention, her mental state improved significantly. She was referred for outpatient follow-up. Patient continued total parenteral nutrition and had performed caregiver training for future home parenteral nutrition. Over the 14-week hospitalization, she gained approximately 7 kg.

**Figure 3:**
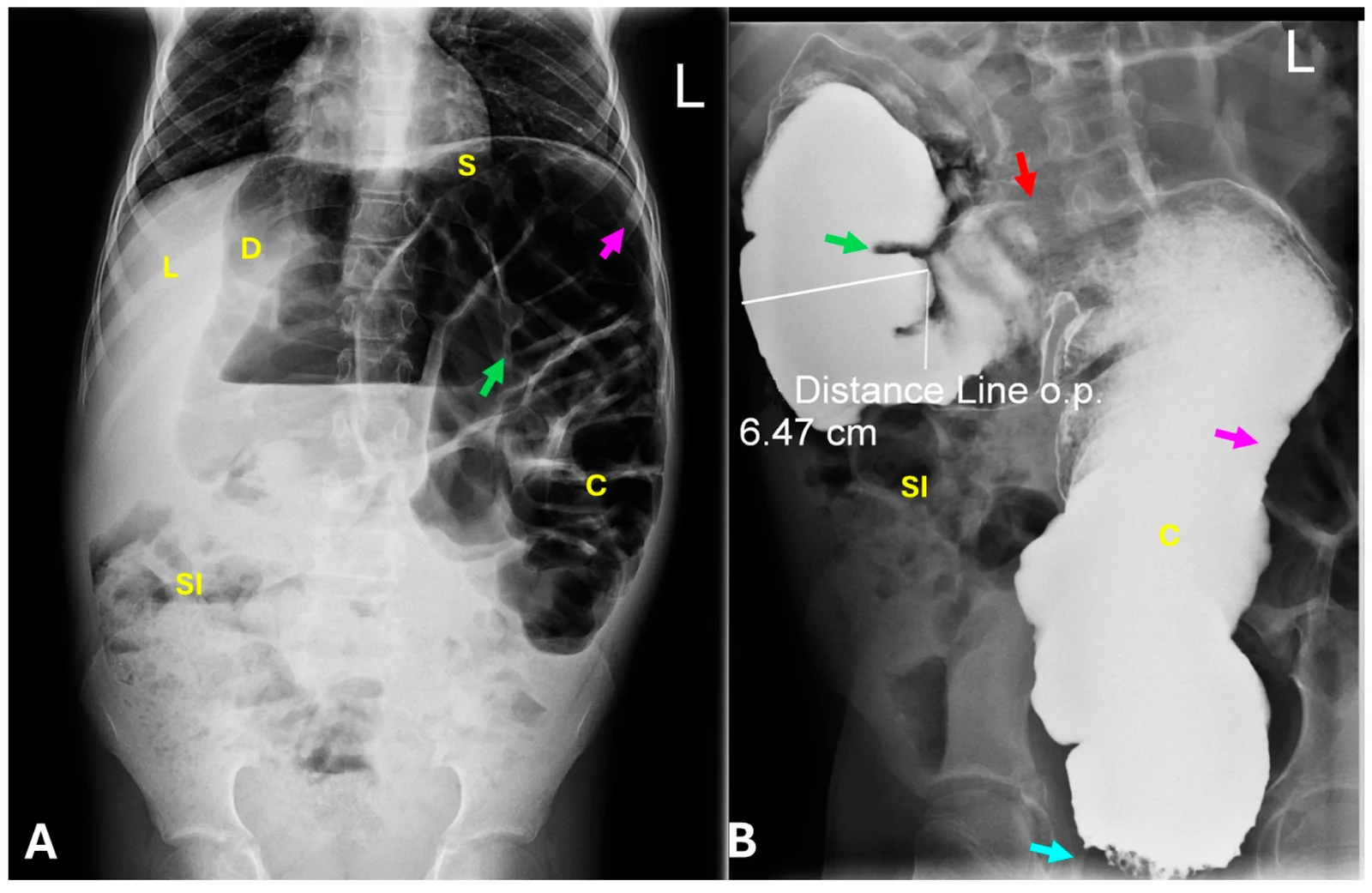
Radiological findings in the 11-year-old boy. (**A**)—standing X-ray; (**B**)—contrast bedside X-ray. S—stomach; D—duodenum; SI—small intestine; C—colon; L—liver. Arrows: magenta—smooth intestine borders (loss of haustration and folds); green—thickened folds; red—segments contract partially; cyan—lack of shrinkage of rectum, abrupt stricture in anal region. The second case was diagnosed with constipation, encopresis (stool staining and incontinence), marasmus, and growth deficiency appearing from the age of six years old. The prolonged five-year diagnosis was caused by COVID-19 epidemic restriction-related worse access to medical help and professional diagnostic tools, guidelines, and low availability of genetic research centers for rare diseases. On admission, the patient complained of disturbances in the rhythm of defecation and a feeling of overflowing in the stomach—at X-ray, the stomach and liver were extremely compressed and pushed to the side. The tests performed revealed protein–energy malnutrition and excluded celiac disease and infections. Abdominal ultrasound revealed dilated loops of the small intestine up to 40 mm, filled with a large amount of gas, and a colon up to 65 mm. An abdominal X-ray revealed massive dilatation of the small intestine, with retention of stool masses in the intestinal lumen (**A**). After Diopeg administration, the boy passed stool 2–3 times weekly. Subsequently, a contrast enema was performed, the result of which may indicate Hirschsprung’s disease with the transition zone on the sigmoid–rectal border (**B**). The result of the anorectal manometry study was characteristic for chronic constipation, but the study did not exclude Hirschsprung’s disease, which rarely might be diagnosed in adults [12]. Uncommon symptoms of Hirschsprung’s disease were partial constriction function and a lack of the shrunken rectal aganglionic part.

**Figure 4:**
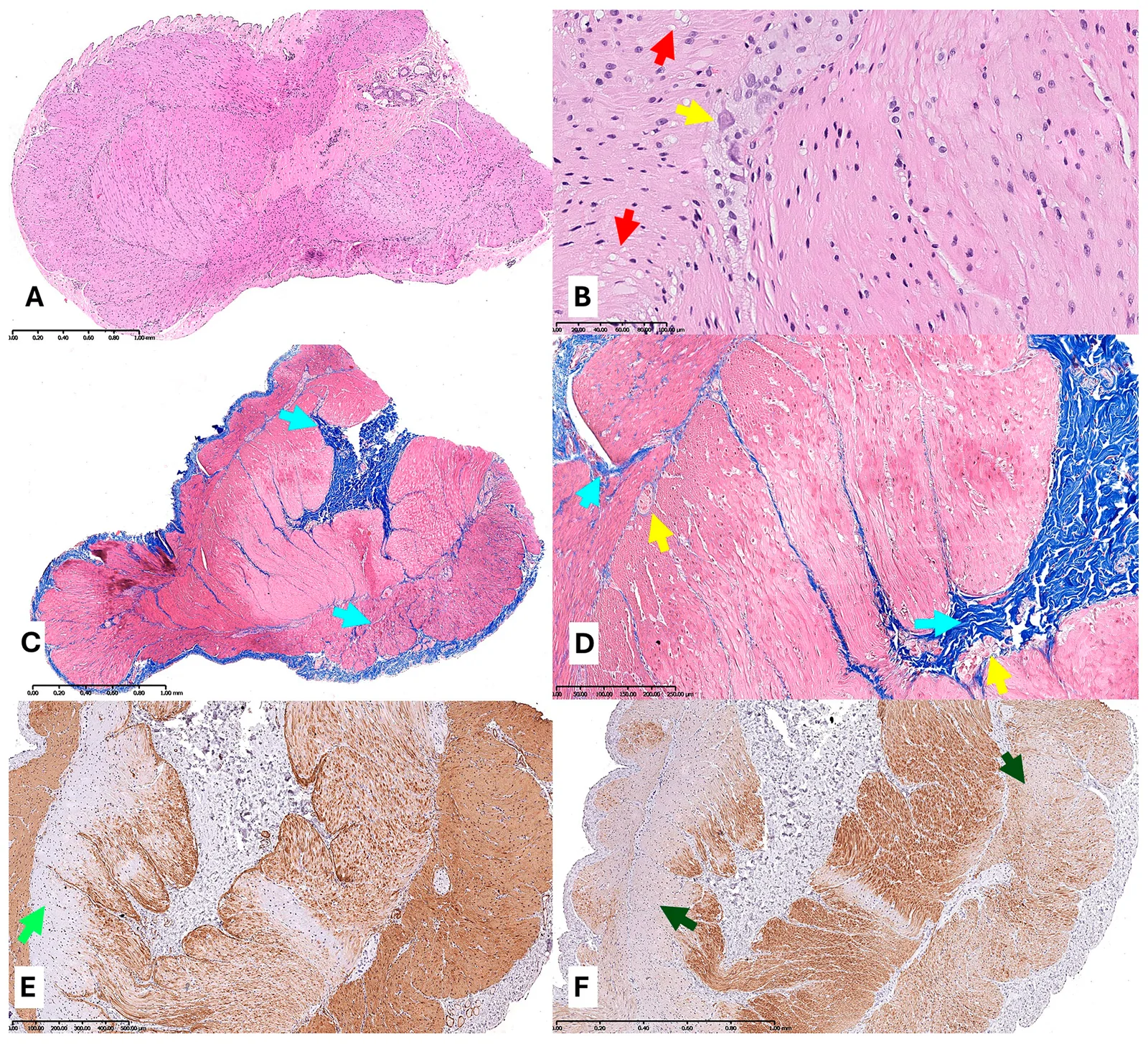
Microphotographs of an ileal biopsy of the 11-year-old boy. (**A**,**B**). HE (×10, ×200). (**C**,**D**). TM (×10, ×100). (**E**) SMA (×40). (**F**) Desmin (×40). Arrows: yellow—ganglion cells of the Myenteric plexus; red—vacuoles; cyan—fibrosis, green—decreased SMA and desmin expression, bright and dark shades, respectively. At age of six, the patient was consulted by a gastroenterologist and dietician—dietary treatment, rectal enemas, and pharmacotherapy of constipation caused clinical improvement. The suction biopsy of the rectum with histopathological examination of mucosal–muscular samples excluded Hirschsprung disease. The symptoms returned, and there was an enlargement of abdominal circumference after six months. The case was consulted in July 2024. Due to diagnostic problems, mapping of the large bowel by the laparoscopic method was performed. Biopsies of muscularis propria were taken from the lower sigmoid, upper sigmoid, splenic flexure, liver flexure, cecum, and ileum. HE staining of ileal muscle biopsies revealed the presence of ganglion cells in the intramuscular and submucosal nerve plexuses, slight thinning, and hypotrophy of both muscle coats and vacuoles in the cell cytoplasm (**A**,**B**). TM staining showed subtle fibrosis in both coats (**C**,**D**). SMA showed a characteristic rimming of the luminal part of the inner muscle coat, weak/no expression in the abluminal part of the inner coat, and normal expression in the outer coat (**E**). Desmin expression was irregularly decreased in different parts of both muscle coats (**F**). Moreover, nerve fibers are poorly visible in S100 staining. Schwann cells and nerve fibers were present in PGP 9.5, in sections from the large intestine, they seemed to be few (in contrast to the control; in the literature, there is no clear pattern or amount of how many of them should be there). Cajal cells are present in DOG1 staining. At most, single scattered leukocytes (CD45+) are found in and around the intramuscular plexuses. The image may correspond to α-SMA deficiency. Diagnosis of VM is frequently delayed, and surgical interventions are often unsuccessful or even detrimental. Myopathies have a high risk for colonic and small bowel volvulus. Regardless of molecular background, VM treatment addresses complications such as electrolyte imbalance, bacterial overgrowth, malnutrition, and constipation [4,7]. The treatment is to alleviate complications; it involves dietary, pharmacological, and surgical interventions. Proper diet, prokinetic agents’ administration, and avoiding drugs that increase the anti-motility effect (e.g., opioids) is substantial. The genetic testing has not been performed due to the improvement of the patient’s condition and the parents’ volition at the time of discharge. Conservative treatment of constipation, prokinetic drugs, and dietary treatment were continued, which provided quality of life. The lack of genetic tests is the main limitation of this manuscript. It would be beneficial for patients to have a pseudo-occlusion genetic panel to fasten the diagnosis and disease management. The current investigation of treatment involves transplantation, artificial stimulation, stem cells, and visceral gene editing [4]. The literature describes world–regional genotype–phenotype occurrence of some special VM types, correlations with severe clinical phenotypes (e.g., ACTG2 mutation with 30% mortality rate), and muscle phenotypic class switching (e.g., in LIX1 mutation). Still, there are unknown mutations and epigenetic changes, and the patient should be referred to dedicated world–regional genetic centers. It is assessed that 60% of VMs had identified genetic mutations. Patients can benefit from genotype–phenotype correlation and specific treatment [4,13,14]. The experiments need VM models of various types [14].

## Data Availability

The original contributions presented in this study are included in the article. Further inquiries can be directed to the corresponding author.

## Abbreviations

The following abbreviations are used in this manuscript:

VM	Visceral Myopathy
HE	Hematoxylin and Eosin
TM	Trichrome-Masson
SMA	Smooth Muscle Actinin Immunohistochemistry

## References

[1] Johnson L.N., Moran S.K., Bhargava P., Revels J.W., Moshiri M., Rohrmann C.A., Mansoori B. (2022). Fluoroscopic Evaluation of Duodenal Diseases. Radiographics.

[2] Rohrmann C.A., Ricci M.T., Krishnamurthy S., Schuffer M.D. (1984). Radiologic and histologic differentiation of neuromuscular disorders of the gastrointestinal tract: Visceral myopathies, visceral neuropathies, and progressive systemic sclerosis. Am. J. Roentgenol..

[3] Thapar N., Saliakellis E., Benninga M.A., Borrelli O., Curry J., Faure C., De Giorgio R., Gupte G., Knowles C.H., Staiano A. (2018). Paediatric Intestinal Pseudo-obstruction: Evidence and Consensus-based Recommendations from an ESPGHAN-Led Expert Group. J. Pediatr. Gastroenterol. Nutr..

[4] Viti F., De Giorgio R., Ceccherini I., Ahluwalia A., Alves M.M., Baldo C., Baldussi G., Bonora E., Borrelli O., Dall’Oglio L. (2023). Multi-Disciplinary Insights from the First European Forum on Visceral Myopathy 2022 Meeting. Dig. Dis. Sci..

[5] Knowles C.H., De Giorgio R., Kapur R.P., Bruder E., Farrugia G., Geboes K., Gershon M.D., Hutson J., Lindberg G., Martin J.E. (2009). Gastrointestinal Neuromuscular Pathology: Guidelines for Histological Techniques and Reporting on Behalf of the Gastro 2009 International Working Group. Acta Neuropathol..

[6] Odze R., Goldblum J. (2023). Surgical Pathology of the GI Tract, Liver, Biliary Tract and Pancreas.

[7] Kapur R.P. (2025). Surgical Pathology of Primary Intestinal Myopathy. Arch. Pathol. Lab. Med..

[8] Finsterer J., Strobl W. (2024). Gastrointestinal Involvement in Neuromuscular Disorders. J. Gastroenterol. Hepatol..

[9] Zhu C.Z., Zhao H.W., Lin H.W., Wang F., Li Y.X. (2020). Latest Developments in Chronic Intestinal Pseudo-Obstruction. World J. Clin. Cases.

[10] Sanders K.M., Ward S.M., Koh S.D. (2014). Interstitial Cells: Regulators of Smooth Muscle Function. Physiol. Rev..

[11] Bianco F., Lattanzio G., Lorenzini L., Mazzoni M., Clavenzani P., Calzà L., Giardino L., Sternini C., Costanzini A., Bonora E. (2022). Enteric Neuromyopathies: Highlights on Genetic Mechanisms Underlying Chronic Intestinal Pseudo-Obstruction. Biomolecules.

[12] Al Jada I., Jabareen M., Alhroub W., Iwaiwi R., Zeidan M., Meshal T. (2025). Delayed diagnosis and management of adult Hirschsprung’s disease: Case report and literature review. Int. J. Surg. Case Rep..

[13] Basilisco G., Marchi M., Coletta M. (2024). Chronic intestinal pseudo-obstruction in adults: A practical guide to identify patient subgroups that are suitable for more specific treatments. Neurogastroenterol. Motil..

[14] Hashmi S.K., Ceron R.H., Heuckeroth R.O. (2021). Visceral myopathy: Clinical syndromes, genetics, pathophysiology, and fall of the cytoskeleton. Am. J. Physiol. Gastrointest. Liver Physiol..

